# A novel federated deep learning scheme for glioma and its subtype classification

**DOI:** 10.3389/fnins.2023.1181703

**Published:** 2023-05-23

**Authors:** Muhaddisa Barat Ali, Irene Yu-Hua Gu, Mitchel S. Berger, Asgeir Store Jakola

**Affiliations:** ^1^Department of Electrical Engineering, Chalmers University of Technology, Gothenburg, Sweden; ^2^Department of Neurological Surgery, University of California, San Francisco, San Francisco, CA, United States; ^3^Department of Neuroscience and Physiology, University of Gothenburg, Gothenburg, Sweden; ^4^Department of Neurosurgery, Sahlgrenska University Hospital, Gothenburg, Sweden

**Keywords:** federated learning, multi-stream FL deep network, deep learning, glioma subtype classification, IDH genotype, domain mapping, extended FedDyn

## Abstract

**Background:**

Deep learning (DL) has shown promising results in molecular-based classification of glioma subtypes from MR images. DL requires a large number of training data for achieving good generalization performance. Since brain tumor datasets are usually small in size, combination of such datasets from different hospitals are needed. Data privacy issue from hospitals often poses a constraint on such a practice. Federated learning (FL) has gained much attention lately as it trains a central DL model without requiring data sharing from different hospitals.

**Method:**

We propose a novel 3D FL scheme for glioma and its molecular subtype classification. In the scheme, a slice-based DL classifier, EtFedDyn, is exploited which is an extension of FedDyn, with the key differences on using focal loss cost function to tackle severe class imbalances in the datasets, and on multi-stream network to exploit MRIs in different modalities. By combining EtFedDyn with domain mapping as the pre-processing and 3D scan-based post-processing, the proposed scheme makes 3D brain scan-based classification on datasets from different dataset owners. To examine whether the FL scheme could replace the central learning (CL) one, we then compare the classification performance between the proposed FL and the corresponding CL schemes. Furthermore, detailed empirical-based analysis were also conducted to exam the effect of using domain mapping, 3D scan-based post-processing, different cost functions and different FL schemes.

**Results:**

Experiments were done on two case studies: classification of glioma subtypes (IDH mutation and wild-type on TCGA and US datasets in case A) and glioma grades (high/low grade glioma HGG and LGG on MICCAI dataset in case B). The proposed FL scheme has obtained good performance on the test sets (85.46%, 75.56%) for IDH subtypes and (89.28%, 90.72%) for glioma LGG/HGG all averaged on five runs. Comparing with the corresponding CL scheme, the drop in test accuracy from the proposed FL scheme is small (−1.17%, −0.83%), indicating its good potential to replace the CL scheme. Furthermore, the empirically tests have shown that an increased classification test accuracy by applying: domain mapping (0.4%, 1.85%) in case A; focal loss function (1.66%, 3.25%) in case A and (1.19%, 1.85%) in case B; 3D post-processing (2.11%, 2.23%) in case A and (1.81%, 2.39%) in case B and EtFedDyn over FedAvg classifier (1.05%, 1.55%) in case A and (1.23%, 1.81%) in case B with fast convergence, which all contributed to the improvement of overall performance in the proposed FL scheme.

**Conclusion:**

The proposed FL scheme is shown to be effective in predicting glioma and its subtypes by using MR images from test sets, with great potential of replacing the conventional CL approaches for training deep networks. This could help hospitals to maintain their data privacy, while using a federated trained classifier with nearly similar performance as that from a centrally trained one. Further detailed experiments have shown that different parts in the proposed 3D FL scheme, such as domain mapping (make datasets more uniform) and post-processing (scan-based classification), are essential.

## 1. Introduction

Deep learning (DL) models require large training datasets to obtain reliable test performance. It has shown promising results for tumor segmentation and classification, in assisting medical diagnostics. However, such studies are mostly focused on a dataset from single cohort/hospital (Zhou et al., 2021), where its size is often small for training a good model and the generalization performance to unseen data from multiple hospitals is poor.

**Figure 1:**
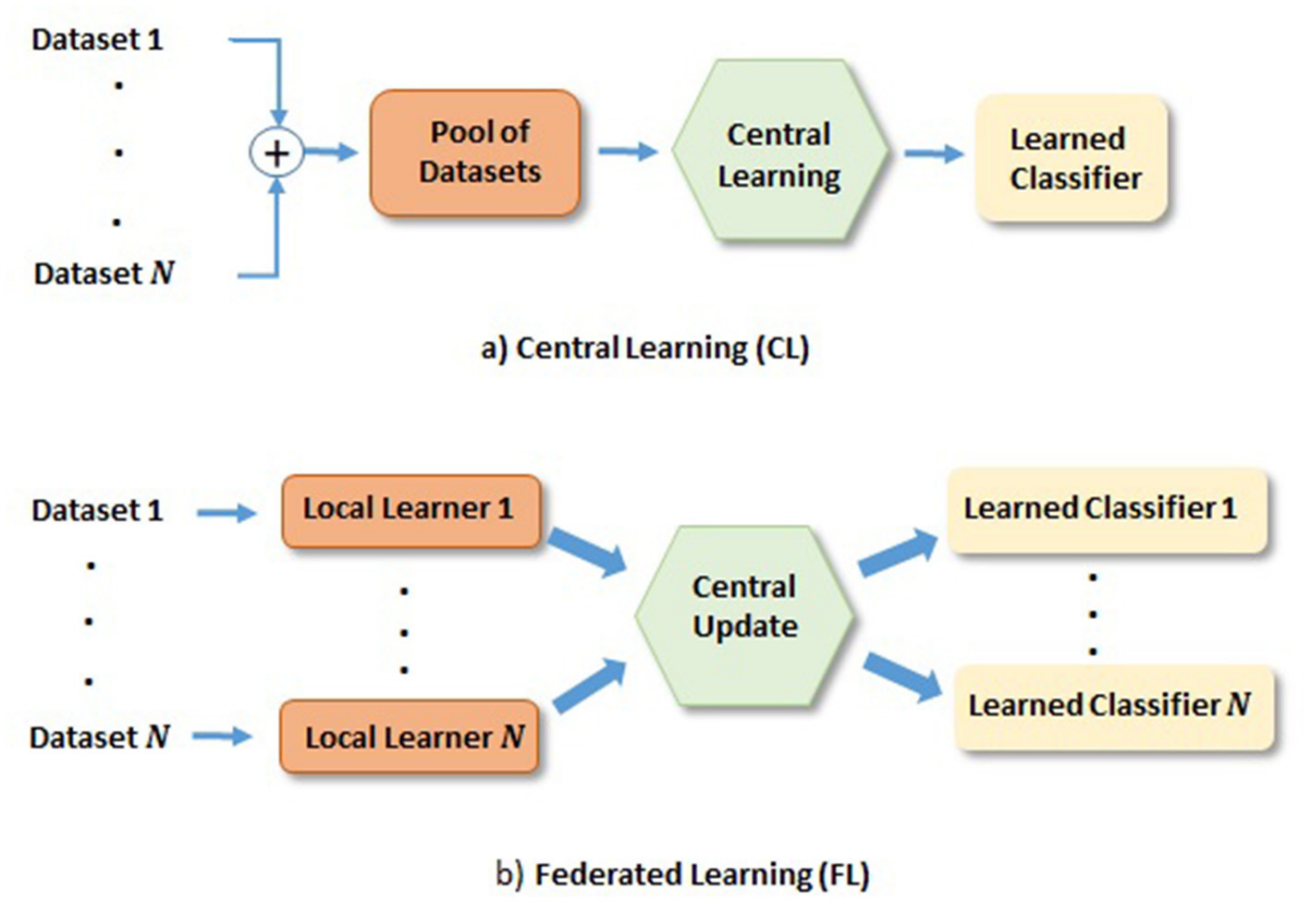
Depiction of FL and CL methods. **(a)** CL: Learning based on centrally shared pool of datasets. **(b)** FL: Learning based on the locally learned models with their private datasets, where local gradients are transferred to the central model for the update and redistribution.

In medical area, one commonly used approach is to share datasets from different hospitals for DL network training. We refer to this approach as the central learning (CL), depicted in Figure 1a, where the datasets from multiple hospitals are combined to train a classifier. However, it has disadvantages, for instance, sharing datasets among hospitals; (1) puts constrain on data privacy and security issues, which many hospitals may not allow. This becomes increasingly difficult when hospitals from multiple countries are involved. (2) Can be complicated by domain shift of datasets from different scanner machines.

Recently, federated learning (FL) has gained much attention as it enables training DL models across hospitals without sharing their datasets, as depicted in Figure 1b. In FL, a set of local models are trained by individual dataset owners in parallel, and the gradients of local model errors are sent to the central model for the update. The central model then sends back the updated model weights to the local model weights before further training. This iteration process continues until the central model converges. One such iteration is referred to as a communication round. Naturally, networks obtained from FL has put demands on communication network, where it is desirable that the required communication rounds are small. It is worth mentioning that heterogeneous data could also prevent FL algorithms from fast converging (Karimireddy et al., 2020a; Khaled et al., 2020). Domain mapping (Khaled et al., 2020) would be desirable in such a scenario. There exist many research directions on FL, e.g., in communication, and in FL network learning, among others. Our study will be focused on latter direction. More specifically, we focus on glioma and its subtype classification based on DL networks trained in the FL fashion.

One of the most common types of brain tumors is glioma. According to World Health Organization (WHO), grade 2 gliomas are referred to as LGG and grade 3 and 4 are referred to as HGG. Based on biopsies from the tumor tissues, gliomas can be further divided into several subtypes (Goodenberger and Jenkins, 2012). WHO has recently revised the glioma classification definition where biomarkers now play more important role in both classification and prognostication. One subtype defined by biomarker is the isocitrate dehydrogenase (IDH) mutation. IDH mutation is found in 70–80% of morphological defined LGG (Parsons et al., 2008) and in ~10% of morphological defined glioblastoma (is now classified as IDH mutated astrocytoma, WHO grade 4). Today IDH mutation provides important information concerning prognosis, response to therapy and clinical decisions (Fuller and Perry, 2005). To identify these subtypes, tissue diagnosis is performed through invasive methods (e.g., biopsy, resection), which comes with inherent risks. Recently, non-invasive methods have been proposed for identifying such information from Magnetic Resonance Images (MRIs) without using biopsy (Buda et al., 2019; Ali et al., 2022; de Dios et al., 2022; Hsu et al., 2022). Though many challenges remain, including, among others, the lack of large amount of annotated training datasets, and data privacy and security issues related to sharing training datasets from different hospitals in different countries.

Several FL approaches have been proposed recently. Among them, one of the basic and most commonly adopted FL methods is Federated Averaging (FedAvg; McMahan et al., 2017). It was reported that FedAvg often suffers from slow convergence if when datasets are heterogeneous. In such a scenario, each local learner pushes the model in a different direction during the training, and the model either does not converge to a global optimum due to client drifting, or, takes excessive number of rounds of communication causing high communication demand. Effort has been made to tackle the heterogeneity data issue in FL. One possible way is to reduce the communications by applying one communication round after several local iterations. Since local optimum in each user is often not consistent with the centralized one (Khaled et al., 2020), further improved approaches were proposed (McMahan et al., 2017; Karimireddy et al., 2020b; Malinovskiy et al., 2020), e.g., by running fewer epochs with each local learner for obtaining a stable though inexact minimization that could perform desirable convergence centrally. Other studies were proposed to deal with heterogeneous data. Karimireddy et al. (2020b) proposed SCAFFOLD that used client variance reduction to correct local updates while assuming that client drift was caused by this variance. Li T. et al. (2020) proposed FedProx to improve the convergence of FedAvg by allowing each local device to train on variable number of local epochs. Wang et al. (2021) proposed FedNova that used variable local updates as well as different local solvers.

FL have also been explored in several medical application. Zerka et al. (2020) proposed a block-chain based platform that combined sequential distributed learning for helping lung cancer diagnosis and claimed similar performance to that of the CL one. Roth et al. (2020) built a FL classification model with improved generalization on seven clinical datasets for breast density classification. Further, only a few FL-based studies were conducted on brain images, e.g., brain tumor segmentation (Li X. et al., 2020; Yi et al., 2020; Nalawade et al., 2022) and brain tumor metastasis identification (Huang et al., 2022). On the other hand, several CL-based approaches were reported for brain tumor classification using datasets such as TCGA and MICCAI. Ge et al. (2019) proposed a classification method for IDH genotype prediction that used GAN for cross modality data augmentation for missing MR modalities. Liang et al. (2018) suggested to use 3D MRI scans with more advanced DenseNets for IDH genotype prediction. Pan et al. (2015) used MR images with some combination operation between multi-phase MRIs, to leverage the learning capability of CNNs for glioma grading. Ge et al. (2018b) proposed to incorporate multi scale features of CNN to extract fine features for glioma grading. However, FL-based brain tumors classification based on glioma and its biomarker-defined subtypes has rarely been reported. One of the main reasons is the lack of large amount of annotated training data since relatively small percentage of tumors are related to the brain, there also exists class imbalance as well as dataset size varies in hospitals. In addition, there is an insufficient amount of annotated brain tumors since both the tumor mask and (newly introduced) molecular-based biomarker are required as the ground truth (GT) labels. Another reason is that different datasets contain MRI scans from different patient cohorts obtained by scanner machines with different acquisition protocols causing a domain shift issue among these datasets.

Motivated by the above issues, this paper proposes a novel and effective glioma and its subtype classification scheme through FL-based training of DL networks on multiple datasets. To the best of our knowledge, this is the first reported successful work on FL-based brain tumor classification on glioma and its molecular subtype from MRIs. The main contributions of this paper include:

- Propose a novel FL-based 3D scheme, consisting of a 2D EtFedDyn classifier, domain mapping on datasets as the pre-processing, and 3D scan-based post-processing to make 3D scan-based prediction on glioma and its subtype.
- Propose a novel FL classifier, EtFedDyn (an extended FedDyn) with the key differences to FedDyn (Acar et al., 2021) on the use of focal loss function (to tackle severe class imbalance) and multi-stream system (for multi-modality MRIs).
- Examine the possibility of replacing CL by FL scheme by comparing the performance of glioma and its subtype prediction from the proposed FL and the corresponding CL schemes.
- Analyze the effect and contributions through empirical tests on domain mapping of datasets, focal loss function over cross-entropy, 3D scan-based post-processing, EtFedDyn over FedAvg classifier including comparisons with several state-of-the-art methods.

The remaining paper is organized as follows. Section 2 describes the proposed FL-based scheme, including the overall description and the details on several key component blocks. Section 3 describes the experiment setup with detailed test results and performance comparisons included. Finally Section 4 concludes the paper.

## 2. Proposed scheme

### 2.1. Overview of the proposed FL scheme

In this section, we propose a novel FL-based glioma and its subtype classification scheme, Our aim is study the feasibility that a FL-based classification scheme may achieve similar performance as that of the corresponding CL one. That implies that each dataset owner may train their dataset jointly with other dataset owners, without providing their dataset to others, yet may obtain a trained classifier that has the similar performance as that of CL one from training all datasets together. To achieve this, several challenging issues are tackled in the proposed scheme, as these issues are especially pronounced in glioma grading and its subtype prediction. These issues include: class imbalances (e.g., between IDH mutation/wild type and between LGG/HGG), domain shift among different hospital datasets, existing of multi-modality MRI data, and potential over-fitting issue when the datasets are small in size, in addition to the concern that privacy and security issues from different hospitals. In this study, these issues are tackled through the use of a novel focal loss instead of cross-entropy as the cost function of FL-based classifier to handle the class imbalances; Domain mapping by CycleGAN on all datasets as the pre-processing; A multi-stream network to learn multi-modality MRI features; and a 2D slice-based EtFedDyn classifier followed by 3D scan-based joint decision as post-processing to mitigate potential over-fitting and consistent decision.

**Figure 2:**
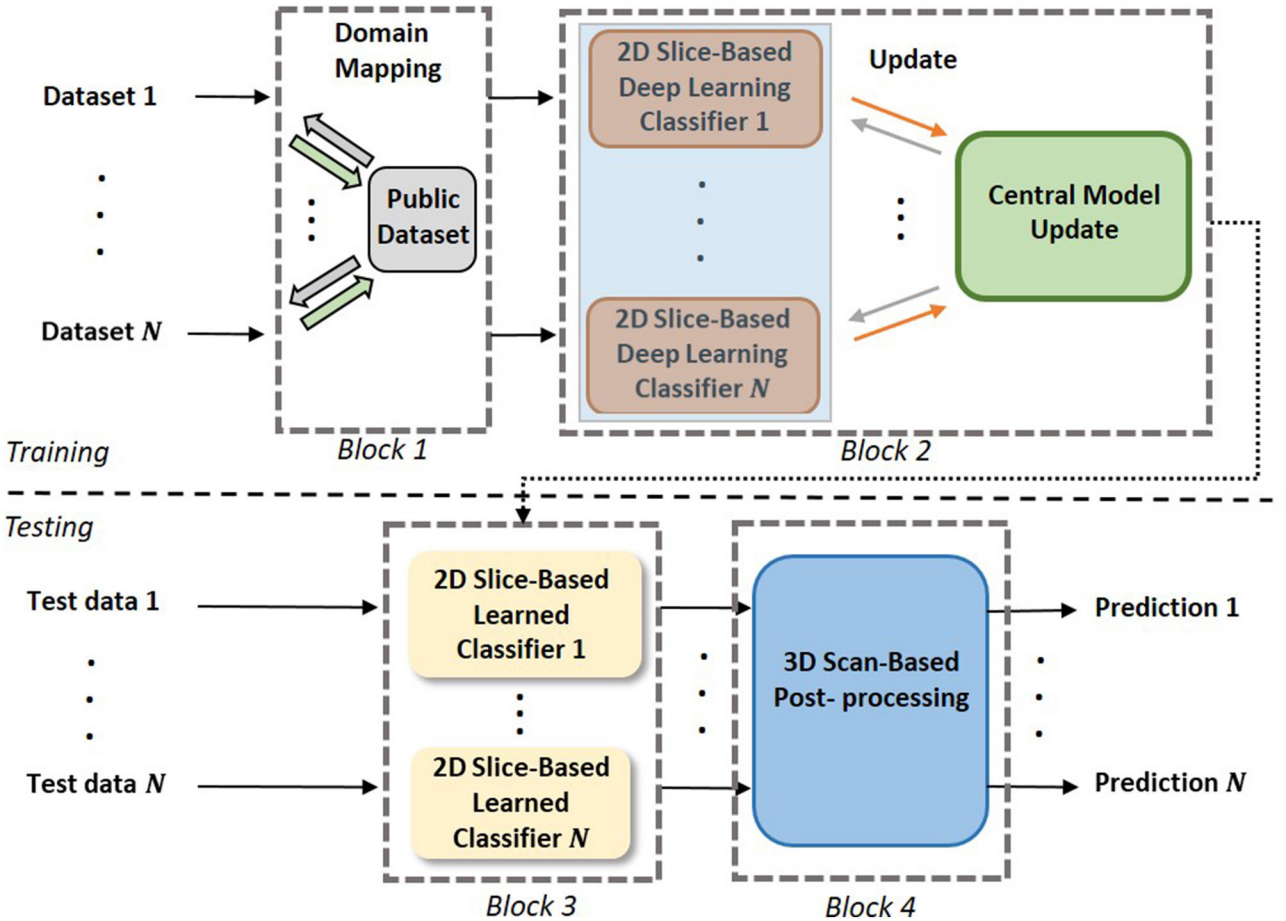
The pipeline of the proposed scheme. **Block 1:** Domain mapping is performed on the datasets. **Block 2:** Training of multi-stream FL-based EtFedDyn classifiers on individual dataset. **Block 3:** Using trained local EtFedDyn model for prediction of glioma and its subtype from MRIs in local test set. **Block 4:** Post-processing on 2D slice-based to predict 3D scan-based patient's diagnosis.

As shown in Figure 2, the proposed 3D FL-scheme consists of a training process (top row) and a testing process (bottom row). In both the training and testing processes, domain mapping of datasets (block 1) is used to make the datasets more uniformly distributed. In the training process, a FL-based 2D slice-based EtFedDyn classifier (block 2) is trained, EtFedDyn is an extension of FedDyn with the focal loss cost function and multi-stream fashion for better network learning on class imbalanced datasets. In the test process, MRI test set, after domain mapping, is fed to EtFedDyn for classification (block 3) whose weights are fixed from the training process. A 3D scan-based post-processing (block 4) is then followed for making a 3D scan-based tumor subtype prediction. In the following section, some essential blocks are explained in details.

### 2.2. Federated learning with focal loss function and multi-stream CNNs

The proposed EtFedDyn classifier is an extended version of FedDyn (Acar et al., 2021). FedDyn is selected as the baseline algorithm since it also includes a regularization term to control the client drift, and local/central iterations to speed up the convergence and reduce the required communication rounds. The proposed EtFedDyn makes the following modification/extension that allows the classifier to tackle the highly imbalanced classes in the training datasets, and to include the use the complimentary information from multi-modality MRIs. We explore focal loss as the cost function for the proposed FL-based 2D classifier (block 2), inspired by the focal loss in Lin et al. (2017) that emphasizes the errors in small classes. We notice that some MRI training classes of datasets are very small due to small percentage of patients with certain glioma grades and biomarker-associated tumor subtypes. Further, a 2D multi-stream convolutional neural network (CNN) is exploited in FL-based classifiers (blocks 2 and 3) for feature learning and classification, for better extracting complementary tumor information from different modalities of MR images.

#### 2.2.1. Focal loss function

The focal loss cost function can be briefly described as a dynamically scaled cross entropy loss that controls the learning on the easy class (i.e., true negatives and true positives) and hard class images (i.e., false positives and false negatives), when the training images have class imbalances. Since images from the major class comprise the main loss and dominate the gradients, focal loss tries to downweigh the confidence in predicting the easy class during the training and allows the model to focus on images from the hard class. To balance the importance between the major and minor classes, a balanced variant of focal loss $L_{focal}\left(p,\hat{p}\right)$ is defined as:


(1) $$\begin{array}{l} L_{focal}\left(p,\hat{p}\right)=-\left[\beta \;{\hat{q}}^{\gamma }p\;\text{log}\left(\hat{p}\right)+\left(1-\beta \right)\;{\left(\hat{p}\right)}^{\gamma }q\;\text{log}\left(\hat{q}\right)\right] \end{array}$$

where $\hat{p}$ and $\hat{q}=\left(1-\hat{p}\right)$ are the predicted probability, *p* and *q* = (1−*p*) are the probability of training images with GT labels, β∈[0, 1] is the weighting factor for major class and (1−β) for minor class, $\hat{q}$ is a modulating factor and γ is a focusing parameter. When γ = 0, (1) becomes the same as the cross-entropy loss. Choosing γ>0 reduces the relative loss for easy class images while putting more focus on hard class images. The parameter values were set empirically as β = 0.25 and γ = 2 in our tests.

#### 2.2.2. Multi-stream 2D FL-based classifier: EtFedDyn

**Figure 3:**
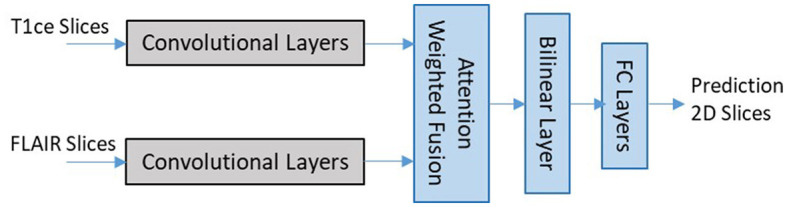
Network architecture for 2D slice-based DL classifier (zoomed in details of block 2 shaded part in Figure 2).

EtFedDyn uses a multi-stream 2D CNN architecture adopted from our previous work Ge et al. (2018a). We use two separate streams of CNNs for learning the glioma subtype features from two MRI modalities (T1ce, FLAIR MRIs), followed by a feature fusion layer (shown in Figure 3).

Each stream of CNN architecture consists of seven convolutional layers with filter sized 3 × 3 in each layer. The last convolutional layer in each stream is related to the modality-specific tumor type features. The outputs from different stream features are then fed to the next layer for fusion and refinement. In the classifier an attention weighted fusion is applied which is different from Ge et al. (2018a), as features from different modality data contribute differently in predicting the glioma and its subtype. Under the fusion strategy, a weighted sum of features is computed such that weights may be learned adaptively based on their modality-specific features. Let *f*_*n*_ denote the vectors of features from the final layer of streams and *w*_*n*_ be the weight matrices for *n* = 1, 2 streams, the fused feature vector is computed by $f=\overset{2}{\underset{n=1}{\sum }}a_{n}f_{n}$, where attention weights *a*_*s*_ for individual modality is computed as:


(2) $$\begin{array}{l} a_{s}=\frac{\text{exp}\left(w_{s}^{T}f_{s}\right)}{\sum_{n=1}^{2}\text{exp}\left(w_{n}^{T}f_{n}\right)} \end{array}$$

where $w_{n}^{T}$ is the transpose of *w*_*n*_. The fused feature layer is then followed by a bilinear layer, two fully connected layers and a final layer for class prediction similar to Ge et al. (2018a).

Let *w*_*c*_ denote the central model weights and *w*_*i*_ the *i*th local model weights, *i* = 1, ⋯ , *N*, where all local models have the same structures. Our objective is to minimize the central model loss *L*_*c*_:


(3) $$\begin{array}{l} L_{c}=\text{arg}\underset{w_{i}}{\min}\left[\frac{1}{N}\overset{N}{\underset{i=1}{\sum }}L_{focal,i}\left(w_{i}\right)\right] \end{array}$$

Since we have only two datasets available, all local models participate for the weight update in each communication rounds. In each communication round *t* = 1, 2, ⋯ , *T*, the central model weights $w_{c}^{t-1}$ at previous round (*t*−1) are used for updating local models, where *i*th local model weights *w*_*i*_, *i* = 1, ⋯ , *N*, are optimized based on the local objective function *L*_*focal, i*_ in 3. This updating process is the same as that of FedDyn baseline method, which can be briefly summarized below. First, the local gradient $g_{i}^{k-1}$ for *i*th model is updated as follows:


(4) $$\begin{array}{l} \begin{array}{c} g_{i}^{k-1}=-\nabla L_{focal,i}\left(w_{i}^{t,k-1}\right)-\nabla L_{focal,i}\left(w_{i}^{t-1}\right)\\ -\alpha \left(w_{c}^{t-1}-w_{i}^{t,k-1}\right) \end{array} \end{array}$$

where *k* is the local epoch, *k* = 1, ⋯ , *K* (*K*=5 in our tests), the last term is the penalty term and α is the regularization parameter. Then, the local model weights *w*_*i*_ are updated using the updated gradient:


(5) $$\begin{array}{l} w_{i}^{t,k}=w_{i}^{t,k-1}-\eta_{i}g_{i}^{k-1} \end{array}$$

where η_*i*_ is the local learning rate. The penalty term $\left(w_{c}^{t-1}-w_{i}^{t,k-1}\right)$ dynamically modifies the local model loss *L*_*focal, i*_, so that the local model would converge similarly as the central model. After last local update epoch *K*, each model weights $w_{i}^{t}$ are then transmitted back to the central model. The averaged local model weights ${\bar{w}}^{t}=\frac{1}{N}\overset{N}{\underset{i=1}{\sum }}w_{i}^{t}$ are used for updating the central model gradient.


(6) $$\begin{array}{l} h_{c}^{t}=h_{c}^{t-1}+\frac{1}{N}\left(w_{c}^{t-1}-{\bar{w}}^{t}\right) \end{array}$$

followed by central model weight update:


(7) $$\begin{array}{l} w_{c}^{t}={\bar{w}}^{t}-h_{c}^{t} \end{array}$$

This process continues until convergence, or a pre-determined maximum communication round *T* is reached.

### 2.3. Domain mapping

MRIs in different datasets from different hospitals/ cohorts were usually obtained from different scanner machines with different machine parameter settings. They often look quite different reflected by the fact that there is a domain shift for MRIs within the same modality. Such differences could be due to the applied magnetic field, the radio pulse sequence frequency, the algorithm that the scanner device follows for image reconstruction and many more. These settings could be different between different hospitals, which may cause heterogeneity in different datasets. To overcome the domain shift, we adapt a domain mapping method from Ali et al. (2020), that uses an unpaired CycleGAN to map MRI data from *i*th dataset *D*^*i*^ to a target dataset *D*^P^, while retaining biomarker-subtype information of gliomas.

The unpaired CycleGAN consists of two generative adversarial networks (GANs), with two generators G_*i*_ and G_P_ and two discriminators D_*i*_ and D_P_. The generators take inputs in parallel from dataset *D*^*i*^ and *D*^P^ and learn to generate the mapped images from ${\hat{D}}^{i}$ and ${\hat{D}}^{P}$, respectively, while the discriminators learn to discriminate between the real and the mapped generated images. The aim is to minimize the objective function given as:


(8) $$\begin{array}{l} L(G_{i},G_{P},D_{i},D_{P})=L_{GAN}(G_{P},D_{P},D^{i},D^{P})\\ +L_{GAN}(G_{i},D_{i},D^{P},D^{i})+{\text{λL}}_{cyc}(G_{i},G_{P}) \end{array}$$

where L_*cyc*_ is the cycle-consistency loss, minimizing L_*cyc*_ ensures the reversible mapping between the two domains and λ is the regularization term. To save the computation, we chose an existing public dataset among the datasets as the target domain D_P_. This mapping is performed by each individual local dataset user independently and the mapped datasets ${\hat{D}}^{i}$ are then used for training the local EtFedDyn classifier.

**Figure 4:**
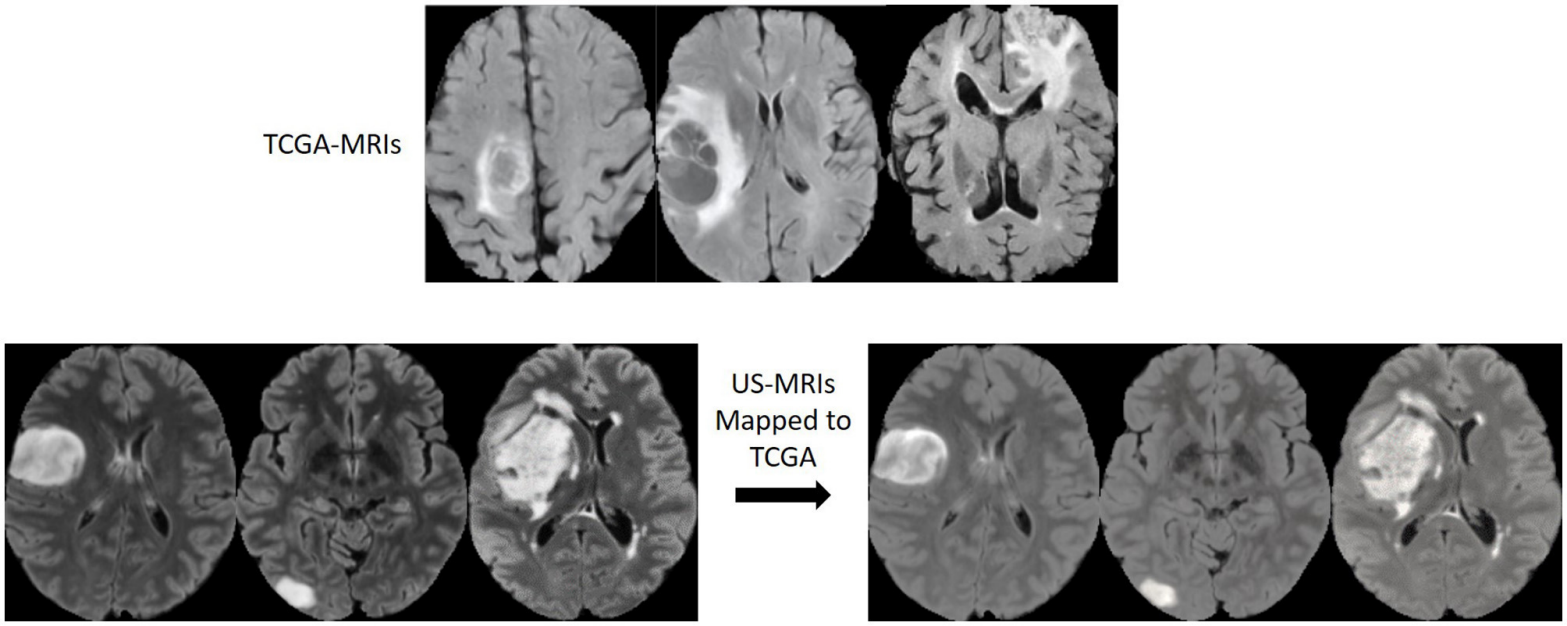
MR image slices before and after the domain mapping. **(Top)** Original FLAIR MR images from TCGA dataset. **(Bottom left)** Original FLAIR MR images from US dataset. **(Bottom right)** Corresponding FLAIR MR images after domain mapping.

Figure 4 shows examples of original and domain mapped MR images from the datasets, so that MR image domains from two different datasets are more similar.

### 2.4. 3D brain scan-based post-processing

**Figure 5:**
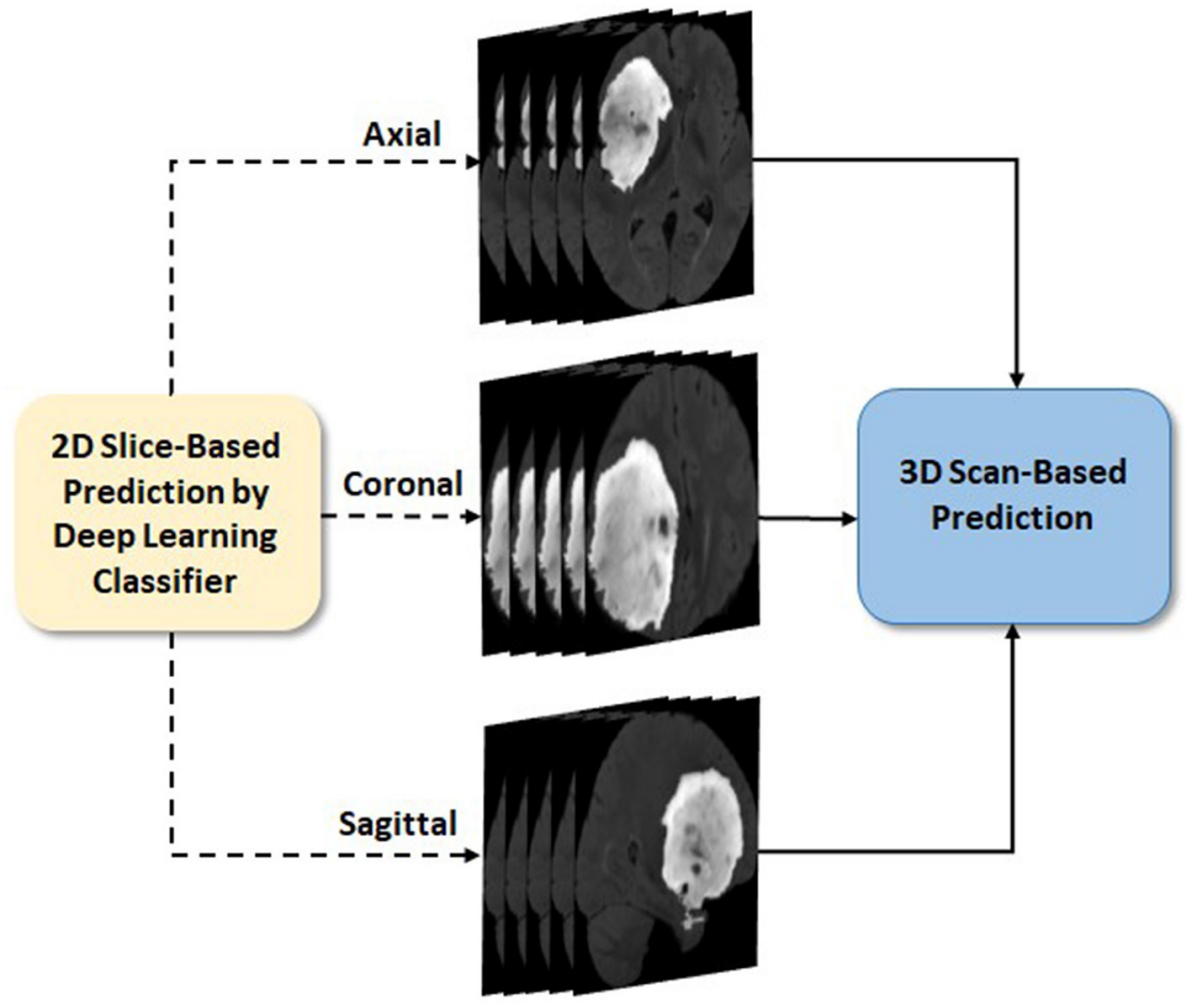
3D scan-based post-processing (Zoom in details of block 4 from Figure 2).

Glioma and its subtype prediction from the FL-based EtFedDyn classifier (Figure 2 blocks 2 and 3) is based on 2D image slices. It is desirable that a consistent prediction could be made based on each individual 3D brain scan for assisting the diagnosis of individual patient. This is done by applying a post-processing block (Block 4 in Figure 2) similar to that in Ge et al. (2020), where the decision for a 3D scan is based on a majority voting-based criterion. The majority of 2D class prediction results would decide the tumor type or biomarker-defined tumor subtype class of a patient (as depicted in Figure 5).

Let *M* be the total number of predicted 2D slice results (from three views) that belong to a patient *O* and *O*_*j*_, (*j* = 1, …, *M*) be the *jth* slice result. The patient *O* as IDH-mutation/LGG class when more than half of the *M* slices are predicted as class 1, otherwise it belongs to IDH wild-type/HGG class:


(9) $$O=\{\begin{array}{ll} 1 & \sum_{j=1}^{M}O_{j}>M/2\\ 0 & \text{Otherwise} \end{array}$$

### 2.5. Pseudocode of the proposed FL scheme

As shown in Algorithm 1, the pseudo codes for the training process of the proposed FL scheme is summarized.

**Algorithm 1:**
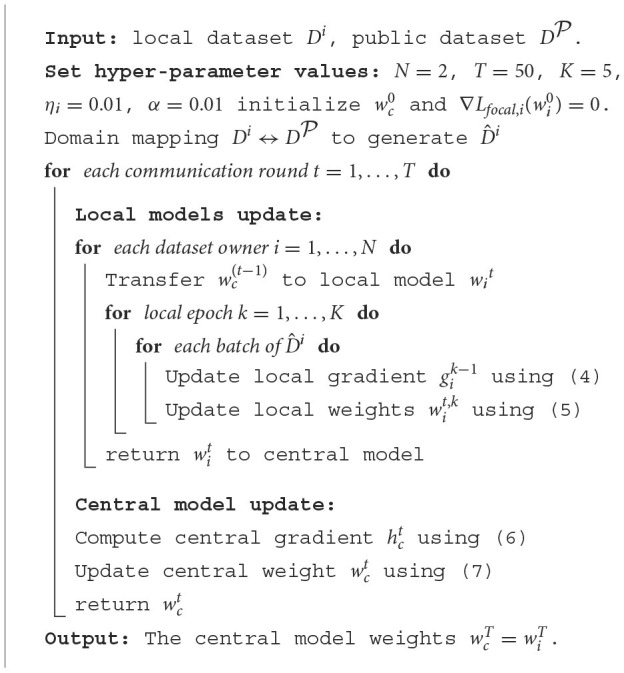
Training process for proposed FL scheme.

### 2.6. Criteria for performance evaluation

The evaluation is performed on the test sets based on the predicted results of glioma or biomarker-defined its subtype classes. All results were averaged in five runs, where each run was performed by patient-wise and random re-partition of training and test sets, retraining and re-testing. Criterion functions; accuracy, sensitivity, and specificity are used for the performance evaluation, defined as follows:


(10) $$\begin{array}{l} \text{Accuracy}=\frac{\text{TP + TN}}{\text{TP + FP + TN + FN}},\;\text{Sensitivity}=\frac{\text{TP}}{\text{TP + FN}},\\ \text{Specificity}=\frac{\text{TN}}{\text{FP + TN}} \end{array}$$

Where we define the IDH mutation/LGG as the positive class, then the definitions of TP, FP, TN, and FN become:

True positive (TP): IDH mutated (or, LGG) class and is predicted as IDH mutated (or, LGG).

False positive (FP): IDH wild-type (or, HGG) class and is falsely predicted as IDH mutated (or, LGG).

True negative (TN): IDH wild-type (or, HGG) class and is predicted as IDH wild-type (or, HGG).

False negative (FN): IDH mutated (or, LGG) class and is falsely predicted as IDH wild-type (or, HGG).

### 2.7. Other implementation issue: data augmentation

A simple data augmentation approach is applied including horizontal flipping and random small angle rotation ( ≤ 10^0^) during online training process.

## 3. Results and comparisons

### 3.1. Setup, datasets, pre-processing

#### 3.1.1. Setup

Experiments on the proposed FL scheme were conducted in Python by using Pytorch library (Paszke et al., 2019) in a workstation with Intel-i7 3.40 GHz CPU, 48 G RAM and an NVIDIA Titan Xp 12 GB GPU. By tuning the network carefully through experiments, different hyper-parameters were selected for the proposed FL and the corresponding CL schemes. We simulated FL scheme for *T* = 50 communication rounds on two case studies (case A and B). On each round, datasets are trained locally for *K* = 5 epochs with a batch size of 50 and learning rate η_*i*_ = 0.01. Weight decay of 0.001 was applied to prevent over-fitting and no learning rate decay was used across communication rounds. The α value was selected as 0.01. For the corresponding CL, we chose batch size as 50, weight decay parameter as 0.0001 and learning rate as 0.001 for 50 iterations. The test performance was then evaluated using the network trained from the last communication round. All experiments were repeated five times on randomly patient-wise partitioned data. Comparisons between the proposed FL and the corresponding CL were performed based on the same data partitions. The setup for training Cycle-GAN in domain mapping was the same as that in Ali et al. (2020).

#### 3.1.2. Datasets

Experiments were conducted on two case studies. Table 1 summarizes the information on all datasets for two case studies. Case A study was conducted on 2 datasets from different data owners, where US dataset was obtained from a university hospital in USA and TCGA dataset was a public dataset from TCGA-GBM (*n* = 101) (Bakas et al., 2017a) and TCGA-LGG (*n* = 66) (Bakas et al., 2017b) for gliomas with IDH mutation/wild-type labels. Unlike TCGA dataset, US dataset consists of only LGG (WHO grade 2) with typical appearances of unenhanced hyperintensity in FLAIR MRIs without contrast enhancement. For TCGA dataset, annotation of tumor boundaries (GT) were available. For US dataset, GT tumor boundaries were semi-manually drawn by medical experts and controlled by senior medical doctors through the help of 3D slicer software (v4.10.2) (Pieper et al., 2004). For case B study, MICCAI dataset was partitioned into two parts (according to patients), as MICCAI 1 and MICCAI 2, as two clients in FL for LGG and HGG classification. The dataset was downloaded from MICCAI BraTS 2017 competition (Menze et al., 2014; Bakas et al., 2017c), consisting of 3D scans with GT tumor annotations.

**Table 1:** Summary of two datasets used in our experiments on case A and B studies.

**Case**	**Dataset**	**No. of patients**	**Train set/**	**# 2D**	**# 2D**
**study**		*class 0/1	**Test set**	**Train set**	**Test set**
A (IDH mut/wt)	TCGA	55/112	134/33	2010	495
	US	68/08	58/18	870	270
B (LGG/HGG)	MICCAI 1	37/105	114/28	1710	420
	MICCAI 2	38/105	115/28	1725	420

IDH mut, IDH mutation; IDH wt, IDH wild-type; LGG, low grade glioma; HGG, high grade glioma.

*Case A: Class 0 is IDH mutation and class 1 is IDH wildtype.

Class B: Class 0 is LGG and class 1 is HGG.

In both case studies, two MRI modalities, i.e., FLAIR (Fluid-Attenuated Inversion Recovery and weighted) and T1ce (T1-contrast enhanced) MRIs in the datasets were used. 2D image slices were used in the experiments instead of 3D scans to mitigate possible over-fitting in DL. Since tumor regions only occupy small parts in the entire brain, five image slices containing the tumor were extracted from each of the three views (axial, sagittal, and coronal). All experiments were conducted by five runs, where the datasets in each run were split randomly patient-wise into two sets: training (80%) and testing (20%) such that no 3D scans of any individual patient from one set would be used in another set. For final performance evaluation, results from five runs were then averaged.

#### 3.1.3. Pre-processing

For US dataset, anatomical images from FLAIR/T1ce MRI scans were registered to 1 mm MNI space template. In addition to this, the bias field correction and skull-stripping were performed using software FSL (Khaled et al., 2020) and ANTs (Malinovskiy et al., 2020). Since MRI scans in TCGA and MICCAI datasets were already pre-processed and co-registered, no pre-processing was added. Further for case A study, domain mapping was applied on 2D image slices. Since TCGA is publicly available, we chose the domain of TCGA dataset as the target domain, instead of creating a new domain. Hence, domain mapping was only needed for the US dataset in case A study. Since case B study uses partitioned datasets from a single dataset, no domain mapping was required. For enhanced learning of tumors, tumor masks were applied where the pixel values outside the tumor were reduced to 1/3 of its original values (Ge et al., 2018a). Moreover, the image size was rescaled to 128 × 128 pixels and pixel values in the images were normalized to [0, 1].

### 3.2. Performance of the proposed FL scheme

#### 3.2.1. Overall performance of the proposed FL scheme

To test the effectiveness of the proposed FL scheme, experiments were conducted on 2 case studies. Table 2 summarizes the 3D scan-based results on the test sets from the proposed FL scheme (see Figure 2). Observing Table 2, for case A study, one can see that a relatively high accuracy (85.46%) was obtained on TCGA test set. Due to imbalance classes in TCGA, higher specificity (89.09%) was obtained for the relatively large class of IDH wild-type, and lower sensitivity (75.18%) for a relatively small class of IDH mutation type. Since US dataset has a much smaller size and extremely imbalance classes, a reasonably good accuracy (75.56%) with sensitivity (78.57% for IDH mutation) and specificity (65% for IDH wild-type, with a very small training set) was obtained. For case B study, higher test accuracy (MICCAI 1: 89.24%, MICCAI 2: 90.72%) was obtained. Here again, due to the imbalance classes in the training sets, there are some differences between sensitivity (MICCAI 1: 79.99%, MICCAI 2: 82.85%) and specificity (MICCAI 1: 92.38%, MICCAI 2: 93.33%) in the two classes. The last column of Table 2 shows, the time required for training an individual dataset during each local epoch. Further, the total number of parameters in the DL network was 76,478,979.

**Table 2:** Performance of proposed 3D scan-based FL scheme (see Figure 2) on the test sets for two case studies.

**Case**	**Dataset**	**3D Acc**.	**3D Sen**.	**3D Spe**.	**Time/Local**
**study**		**%(∣σ∣)**	**%(∣σ∣)**	**%(∣σ∣)**	**Epoch (sec.)**
A (IDH mut/wt)	TCGA	85.46 (3.53)	78.18 (7.27)	89.09 (4.63)	137.39
	US	75.56 (2.72)	78.57 (6.38)	65.00 (12.25)	59.24
B (LGG/HGG)	MICCAI 1	89.28 (2.26)	79.99 (6.99)	92.38 (3.81)	116.75
	MICCAI 2	90.72 (1.75)	82.85 (5.71)	93.33 (2.34)	117.30

Acc, accuracy; Sen, sensitivity; Spe, specificity.

#### 3.2.2. Comparison of proposed FL vs. corresponding CL scheme

The aim of this part of the study is to examine whether one may replace a CL scheme by a FL scheme, such that individual dataset owner may train their DL network while retaining their dataset without loosing the privacy. More specifically, we would like to examine the performance degradation by using FL scheme in place of the corresponding CL scheme. For FL scheme, the performance is computed by combining the test sets from two datasets. For CL scheme, the datasets were combined (neglecting the privacy concerns) before using them for training and testing. For fair comparison, we used the same data partitions of the datasets, where the CL network was corresponding to the FL network in terms of DL network architecture, also the same domain mapping (case A only) and 3D post-processing. Table 3 summarizes the average performance on the test sets from the proposed FL scheme and the corresponding CL one. One may observe that the proposed FL has a small performance degradation of about 1.17% in average test accuracy on case A study and 0.83% on case B study.

**Table 3:** Performance comparison on 3D scan-based test results of the proposed FL vs. its corresponding CL scheme on 2 case studies (see Figure 2 with block 4).

**Case**	**Proposed FL**	**Corresponding CL**	**Performance**
**study**	**% (∣σ∣)**	**% (∣σ∣)**	**difference**
A	81.96 (2.88)	**83.13 (2.94)**	−1.17
B	89.88 (1.68)	**90.71 (1.33)**	−0.83

The bold numbers indicate relatively higher values.

#### 3.2.3. Performance of 2D slice-based results and effect of post-processing

**Figure 6:**
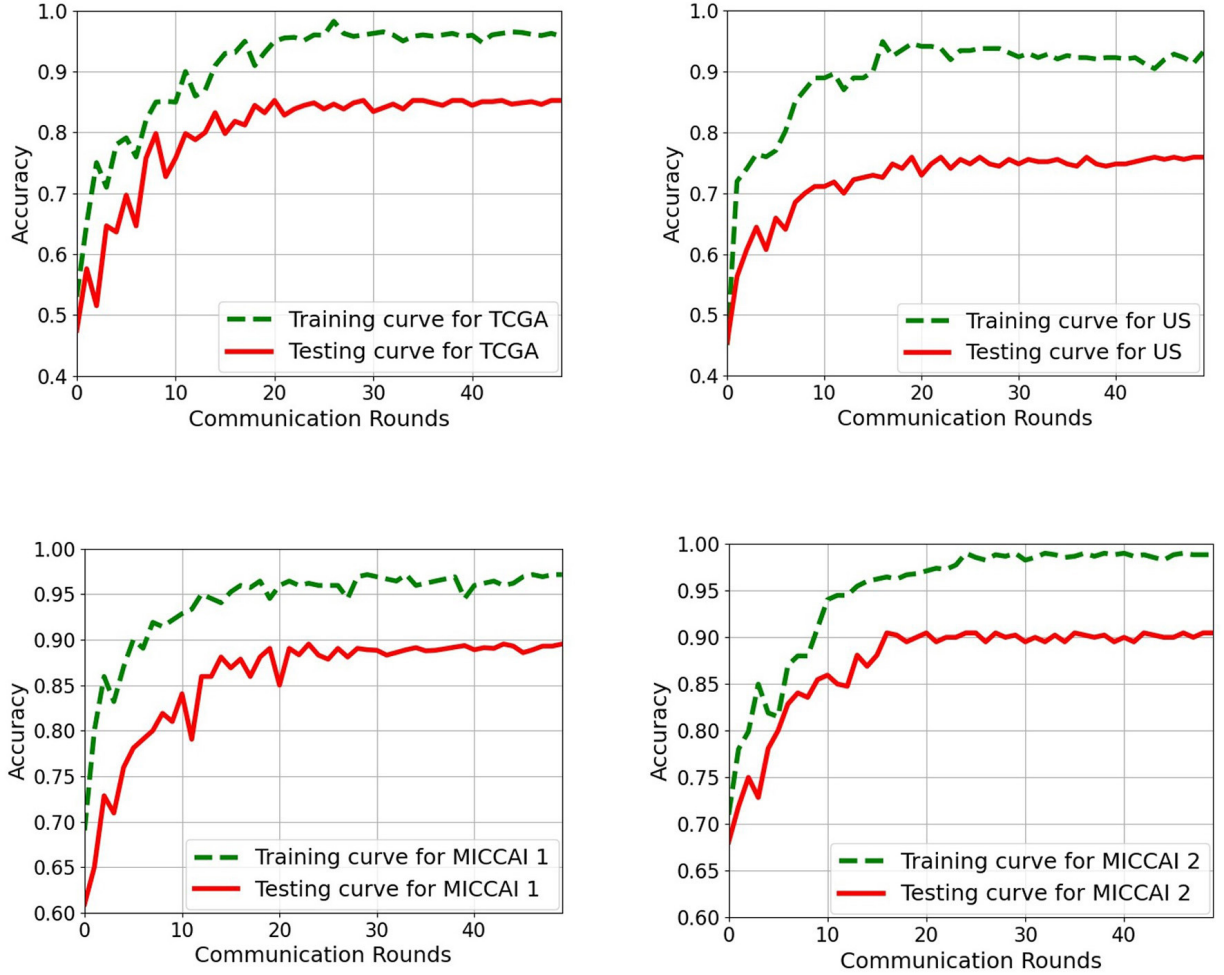
Training and testing curves of the proposed EtFedDyn scheme on both case studies for a single run. **(Top)** Case A study on TCGA and US datasets. **(Bottom)** Case B study on MICCAI 1 and MICCAI 2. **Green curve:** Training curve. **Red curve:** Testing curve.

The aim of this part is to examine the performance of the 2D EtFedDyn classifier (i.e., Figure 2 without using block-4) and to find the effect of 3D post-processing (i.e., Figure 2 with block-4). The EtFedDyn performance from the proposed FL scheme on the test sets are shown in Table 4 and the corresponding training and testing curves are shown in Figure 6.

**Table 4:** Performance of EtFedDyn classifier (see Figure 2 without using block 4) on the test sets from two datasets.

**Case study**	**Dataset**	**2D Acc**.	**2D Sen**.	**2D Spec**.
		***%*(∣σ∣)**	***%*(∣σ∣)**	***%*(∣σ∣)**
A (IDH mut/wt)	TCGA	83.35 (2.94)	75.39 (7.40)	87.33 (4.00)
	US	73.33 (3.38)	76.76 (6.62)	61.33 (8.59)
B (LGG/HGG)	MICCAI 1	87.47 (1.42)	79.05 (6.57)	90.28 (3.29)
	MICCAI 2	88.33 (1.93)	80.38 (6.61)	90.98 (2.59)

Acc, accuracy; Sen, sensitivity; Spe, specificity.

From Tables 2, 4, one can calculate the values in Table 5, which indicate the effect of 3D post-processing. Observing Table 5, one can see that 3D scan-based post-processing has significantly improved the performance of the proposed scheme on the test sets (case A: by 2.11%, 2.23% for TCGA and US datasets, Case B: by 1.81%, 2.39% for MICCAI 1 and MICCAI 2).

**Table 5:** Effect of 3D-based post-processing on the test accuracy for two case studies.

**Case study**	**Dataset**	**Acc. improvement (%)**
A (IDH mut/wt)	TCGA	2.11
	US	2.23
B (LGG/HGG)	MICCAI 1	1.81
	MICCAI 2	2.39

In the rest of subsections below, the performance analysis was conducted on the FL-based 2D EtFedDyn classifier (Blocks 2, 3 of Figure 2) on test sets.

#### 3.2.4. Performance comparison of proposed EtFedDyn by using different loss functions

The aim of this part is to examine the effect of using different loss functions in the EtFedDyn classifier (i.e., loss function in blocks 2 and 3 of Figure 2). Observing that the high class imbalance in the brain tumor and its subtype data, focal loss function was applied in order to enhance the performance.

**Figure 7:**
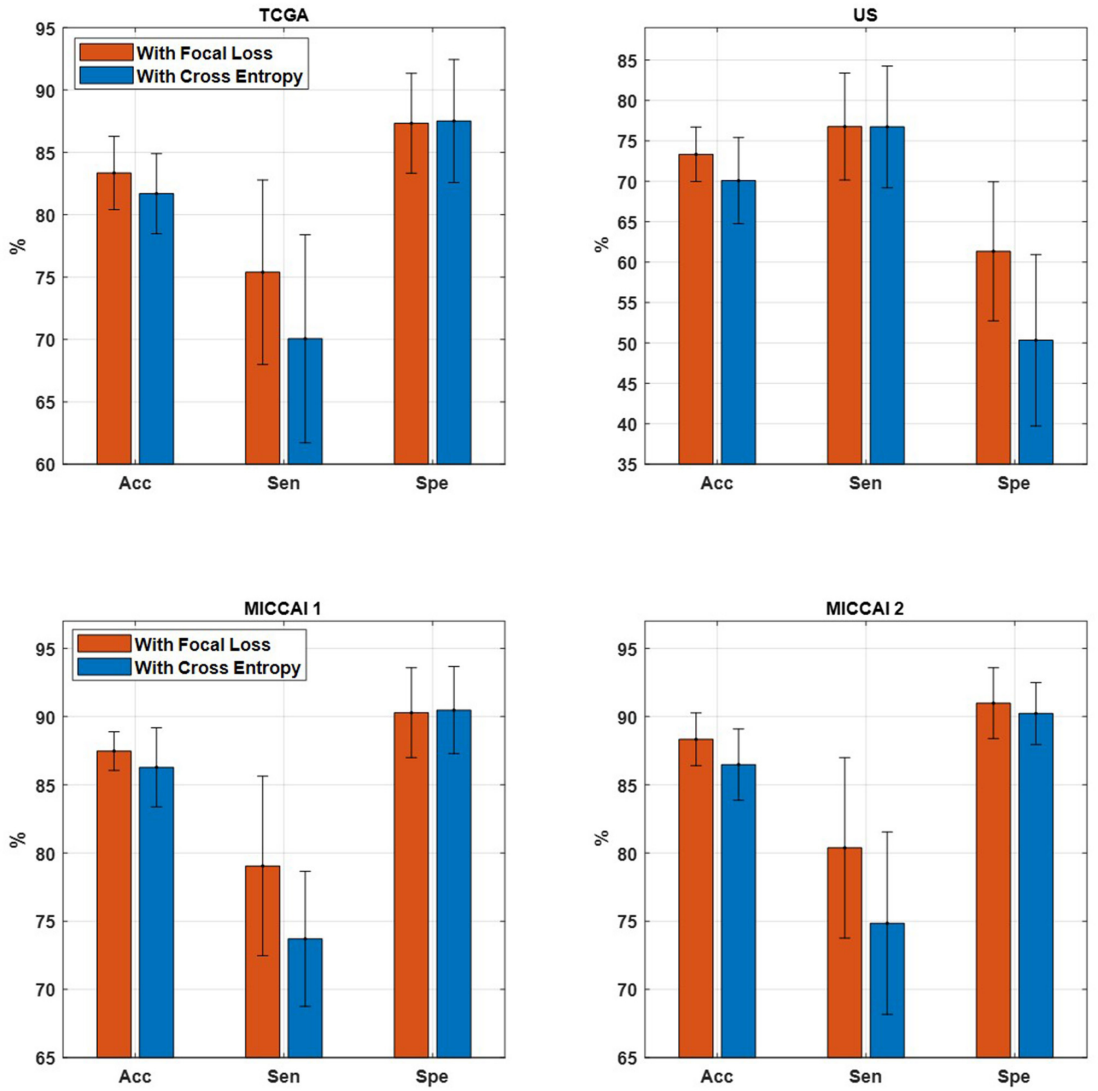
Comparison of proposed EtFedDyn on accuracy, sensitivity, and specificity. **(Top)** TCGA and US datasets for case A study. **(Bottom)** MICCAI 1 and MICCAI 2 datasets for case B study. **Red bar:** With focal loss *L*_*focal*_. **Blue bar:** With cross-entropy loss *L*_*ce*_.

Table 6 shows the performance of EtFedDyn classifiers from using focal loss *L*_*focal*_ and cross-entropy *L*_*ce*_ in the proposed scheme. Observing Table 6 and Figure 7, one can see that the test accuracy from using focal loss function is improved over that from cross-entropy one (case A: by 1.66%, 3.25% for TCGA and US datasets, case B: by 1.19%, 1.85% for MICCAI 1 and MICCAI 2) all with decreased standard deviation. Further, one may observe that using focal loss has improved sensitivity in TCGA and MICCAI dataset, and specificity in US dataset, respectively.

**Table 6:** Performance comparison of proposed EtFedDyn classifier by using focal loss *L*_*focal*_ and cross-entropy loss *L*_*ce*_ on case studies.

**Case**	**Dataset**	**Loss**	**Acc**.	**Sen**.	**Spe**.
**study**		**Func**.	***%*(∣σ∣)**	***%*(∣σ∣)**	***%*(∣σ∣)**
A	TCGA	*L*_*fool*_	**83.35 (2.94)**	**75.39 (7.40)**	87.33(4.00)
IDH mut/wt		*L*_*ce*_	81.69 (3.21)	70.06 (8.34)	**87.51 (4.93)**
	US	*L*_*focal*_	**73.33 (3.38)**	**76.76 (6.62)**	**61.33 (8.59)**
		*L*_*ce*_	70.08(5.33)	76.72 (7.53)	50.33 (10.61)
B	MICCAI 1	*L*_*fool*_	**87.47 (1.42)**	**79.05 (6.57)**	90.28 (3.29)
LGG/HGG		*L*_*ce*_	86.28(2.89)	73.71 (4.95)	**90.47 (3.19)**
	MICCAI 2	*L*_*focal*_	**88.33 (1.93)**	**80.38 (6.61)**	**90.98 (2.59)**
		*L*_*ce*_	86.48 (2.61)	74.85(6.69)	90.22(2.27)

Acc, accuracy; Sen, sensitivity; Spe, specificity.

The bold numbers indicate relatively higher values.

#### 3.2.5. Comparison of 2 FL schemes: proposed EtFedDyn vs. corresponding FedAvg

This part is aimed at comparing the test performance by using two different FL schemes. More specifically, we compare the performance of proposed EtFedDyn classifier and the corresponding FedAvg classifier (with focal loss function) in terms of classification accuracy and the convergence speed. Case A study uses domain mapped data in both the methods.

Table 7 shows the performance on the test sets from the two FL schemes on two case studies. Observing the results in Table 7, one can see that the proposed EtFedDyn classifier has obtained better test accuracy (case A: 83.35%, 73.33% for TCGA and US datasets, case B: 87.47%, 88.33% for MICCAI 1 and MICCAI 2) than the corresponding FedAvg classifier (case A: 82.30%, 71.78% for TCGA and US, case B: 86.24%, 86.52% for MICCAI 1 and MICCAI 2).

**Table 7:** Performance comparison between EtFedDyn classifier and the corresponding FedAvg (with *L*_*focal*_) classifier on test sets, in terms of classification accuracy and convergence speed.

**Case study**	**Dataset**	**EtFedDyn**	**FedAvg**
A (IDH mut/wt)	TCGA	**83.35 (2.94)**	82.30 (2.61)
	US	**73.33 (3.38)**	71.78 (3.95)
B (LGG/HGG)	MICCAI 1	**87.47 (1.42)**	86.24 (1.86)
	MICCAI 2	**88.33 (1.93)**	86.52 (1.32)

The bold numbers indicate relatively higher values.

**Figure 8:**
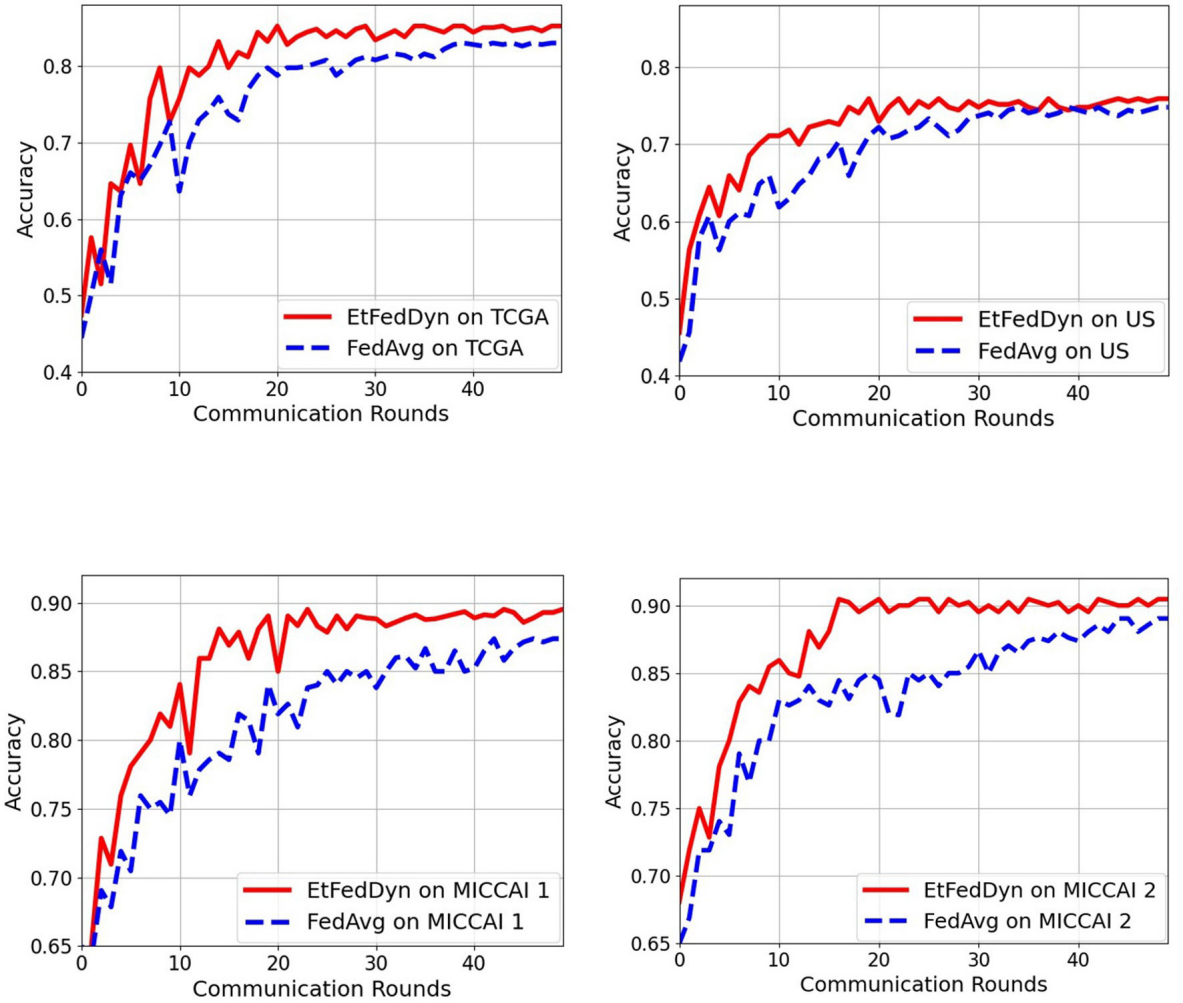
Comparison of communication rounds required in the proposed EtFedDyn and the FedAvg (with focal loss) scheme. The test curves during training for a single run. **(Top)** The convergence curves for TCGA and US datasets in case A study. **(Bottom)** The convergence curves for MICCAI 1 and MICCAI 2 in case B study. **Red curve:** For EtFedDyn. **Blue curve:** For FedAvg (with focal loss).

Figure 8 shows the convergence curves as a function of communication rounds for the two FL classifiers during the training processes on two case studies. From the curves, one can see that the proposed EtFedDyn classifier converges faster hence required less communication rounds (EtFedDyn converged after 10–20 rounds, FedAvg converged after 30–40 rounds) for reaching the convergence on test performance.

It is worth noting, that the performance comparison between the proposed EtFedDyn and the original FedDyn (with inclusion of 2 streams, added domain mapping and 3D post processing similar to that shown in Figure 2 has also been compared, see the results in Table 6 in Section 3.2.4.

#### 3.2.6. Effect of domain mapping

This analysis is aimed at examining the effect of applying domain mapping. Since only case A study required domain mapping (while in case B, two partitioned datasets were obtained from the same MICCAI dataset, hence no domain mapping was required), the study was only conducted on case A study. Domain mapping is aimed at making the classifiers less affected by the data made from different scanner settings and/or from different equipment in hospitals. Experiments were conducted using the proposed FtFedDyn, with and without applying domain mapping (i.e., with/without block-1 in Figure 2). Table 8 shows the performance on the test sets using EtFedDyn with and without domain mapping.

**Table 8:** Comparison of EtFedDyn classifier test results in case A study, to examine the effect of domain mapping for proposed FL scheme on two datasets.

**Domain mapping**	**TCGA**	**US**
With	**83.35 (2.94)**	**73.33 (3.38)**
Without	82.95 (2.54)	71.48 (2.93)
Difference	0.4 (0.4)	1.85 (0.45)

The bold numbers indicate relatively higher values.

One can see from Table 8 that adding domain mapping block in Figure 2 has moderately improved the test accuracy (0.4% for TCGA, 1.85% for US dataset) with a slight increase of standard deviations. The performance improvement was relatively small since the baseline FedDyn has already contained a regularization term to handle data heterogeneity. It is worth mentioning that for the FedAvg (with focal loss) classifier more significant improvement on test accuracies were obtained (with +2.79% for TCGA and +4.45% for US dataset).

#### 3.2.7. Comparison to state-of-the-art methods

The performance comparison to some of the existing methods that have used the same datasets as the proposed scheme are shown in Table 9. All methods in Table 9 used for comparison with the proposed scheme, have employed CL approaches. Therefore, results from Liang et al. (2018), Ge et al. (2019), and Ali et al. (2020) for case A and the results from Pan et al. (2015) and Ge et al. (2018b) for case B can only be used as a performance indication, especially when datasets were not exactly the same in some cases. Observing Table 9, it is shown that the proposed scheme has better performance than Liang et al. (2018), Ge et al. (2019), and Ali et al. (2020) for predicting IDH mutation and wild-type glioma subtypes and also better performance than Pan et al. (2015) and Ge et al. (2018b) for LGG and HGG classification. These comparisons have also indicated that the proposed scheme is effective with the performance comparable to the state-of-the-art methods with the additional FL advantage on preserving dataset privacy/security.

**Table 9:** Comparison of two case studies from the proposed scheme with some existing methods.

**References**	**Method**	**Dataset**	**No. of patients (IDH**	**Test**
			**mut/wt)**	**Acc. (%)**
**Case A (IDH mut/wt)**				
Ge et al. (2019)	CL	TCGA	55/122	81.03
Liang et al. (2018)	CL	TCGA	55/112	84.60
Ali et al. (2020)	CL	US+France	137/24	72.38
Proposed	FL	US	68/08	**75.56**
		TCGA	55/122	**85.46**
**References**	**Method**	**Dataset**	**No. of patients (LGG**	**Test**	
			**/HGG)**	**Acc. (%)**
**Case B (LGG/HGG)**				
Pan et al. (2015)	CL	MICCAI	25/188	73.33
Ge et al. (2018b)	CL	MICCAI	75/210	89.47
Proposed	FL	MICCAI 1	75/210	**89.88**
		MICCAI 2		

The bold numbers indicate relatively higher values.

### 3.3. Discussion and future work

Some insights obtained from our experimental results using the proposed scheme are:

- The proposed 3D brain scan-based FL scheme has obtained competitive performance as comparing to the corresponding CL approach. It has only a slight decrease of 1.17% for glioma subtype and 0.83% for glioma LGG/HGG classification in terms of average test accuracy, while enable hospitals maintaining their own datasets, where privacy/security issues may be tackled through FL.
- The proposed EtFedDyn with focal loss function has improved the test performance, by overweighing errors from small data class and alleviating the class data imbalances in the training sets (case A: +1.66%, +3.25% for glioma IDH subtypes and case B: +1.19%, +1.85% for glioma LGG/HGG in our tests).
- Domain mapping is useful to handle datasets consists of scans from different cohorts/hospitals with different scanners/scanner settings. For EtFedDyn (already contains regularization term for data heterogeneity), moderated improvement is expected (0.4%, 1.85% increase in test accuracy in our tests). For Basic FedAvg classifier, the improvement is expected relatively large (2.79%, 4.45% in our tests).
- EtFedDyn classifier has a fast convergence and better classification accuracy on the test sets than the corresponding FedAvg classifier (improved by 1.05%, 1.55% for glioma IDH subtypes in case A study and 1.23%, 1.81% for glioma LGG/HGG in case B study in our tests, and also with ~50% faster convergence speed).
- Post-processing offered a 3D scan-based patient level decision on glioma subtypes, while being relatively simple, it offers relatively significant gains in performance (test accuracy improved by 2.11%, 2.23% for glioma subtypes in case A study and 1.81%, 2.39% for glioma LGG/HGG grades in case B study tests).
- Comparison of performance with several state-of-the-art methods has indicated that the proposed FL scheme has reached comparable performance to those of some of the existing methods based on CL approach.

### 3.4. Limitations and future work

The datasets on glioma types and their biomarker defined subtypes have been mostly found in a small/moderate size from different hospitals in different countries. Hence, handling data privacy constraint becomes pronounced issue. Our current work was only conducted on two datasets. Future work will be on using more hospital datasets for testing the performance of the proposed FL scheme and to evaluate its possibility of replacing the corresponding CL approach.

## 4. Conclusion

The proposed 3D brain scan-based FL scheme, consisting of a novel 2D FL classifier (EtFedDyn), in combination with domain mapping as pre-processing and scan-based decision as post-processing, is shown to be effective in providing good test performance on classifying glioma subtypes (IDH mutation and IDH wild type) on two datasets and on classifying glioma grades (LGG/HGG) on a single dataset. Comparing with the corresponding CL approach, the proposed scheme has provided a competitive performance with only a small drop in average test accuracy (−1.17%, −0.83%), while offered the advantage of maintaining data privacy where each hospital may train its own dataset on its local DL network. Detailed empirical analysis was also performed to verify the contributions from individual parts of the scheme, including cost functions, FL schemes, domain mapping and post-processing, among others. Comparisons with several existing state-of-the-art CL methods, the proposed FL-based method EtFedDyn still maintains competitive performance. Comparisons with two existing FL approaches (FedAvg and FedDyn) have also shown improved test performance. Limitations and future work were also discussed.

## Data availability statement

The original contributions presented in the study are included in the article/supplementary material, further inquiries can be directed to the corresponding author.

## Ethics statement

The studies involving human participants were reviewed and approved by Ethical Committee of Western Sweden (Dnr: 702-18) and of institutional review boards of participating centers. The patients/participants provided their written informed consent to participate in this study.

## Author contributions

MA and IG developed the method, designed experiments, and did the manuscript writing. MA performed the experiments. MB provided US dataset. US data was annotated under supervision of AJ who also provided medical background, contributed in exchange of ideas, and paper drafting. All authors have read and approved the final draft.
